# Identification of mesenchymal-to-epithelial transition during heart regeneration through genetic lineage tracing

**DOI:** 10.1186/s13287-023-03391-8

**Published:** 2023-06-14

**Authors:** Zibei Gao, Zhengkai Lu, Jinyan Meng, Chao-Po Lin, Hui Zhang, Juan Tang

**Affiliations:** 1 School of Life Science and Technology, ShanghaiTech University, Shanghai, 201210 China; 2 Institute for Regenerative Medicine, Shanghai East Hospital, Frontier Science Center for Stem Cell Research, School of Life Science and Technology, Tongji University, Shanghai, 200092 China

**Keywords:** Fibroblasts, Epicardium, Lineage tracing, Regeneration, Apical resection

## Abstract

The epicardium is the important outermost mesothelial/epithelial layer of the heart that serves as a signaling center for cardiac development and repair. During heart development, epicardial cells undergo a process known as epithelial-to-mesenchymal transition to form diverse mesenchymal cell lineages, such as fibroblasts, coronary vascular smooth muscle cells, and pericytes. However, it is not clear whether the reverse process, mesenchymal-to-epithelial transition (MET), takes place in the mammalian heart. In this study, we performed apical resection on neonatal hearts and used *Fap-CreER;Ai9* labeling to track activated fibroblasts in the injured cardiac regions. We found that these fibroblasts underwent MET to generate epicardial cells during heart regeneration. To our knowledge, this is the first report of MET occurring in vivo during heart development and regeneration. Our findings suggest that it is feasible to directly convert fibroblasts into epicardial cells, providing a novel approach to generate epicardial cells.

**Supplementary Information:**

The online version contains supplementary material available at 10.1186/s13287-023-03391-8.

## To the editor,

The epicardium is a multipotent cardiac progenitor tissue and a signaling center for cardiac development and regeneration, comprising the outermost mesothelial/epithelial layer of the heart [1]. During heart development, epicardial cells undergo epithelial-to-mesenchymal transition (EMT) to form diverse mesenchymal cell lineages, such as fibroblasts, coronary vascular smooth muscle cells, and pericytes [2]. However, it is unclear whether the reverse process, mesenchymal-to-epithelial transition (MET), occurs in mammalian hearts.

**Fig. 1:**
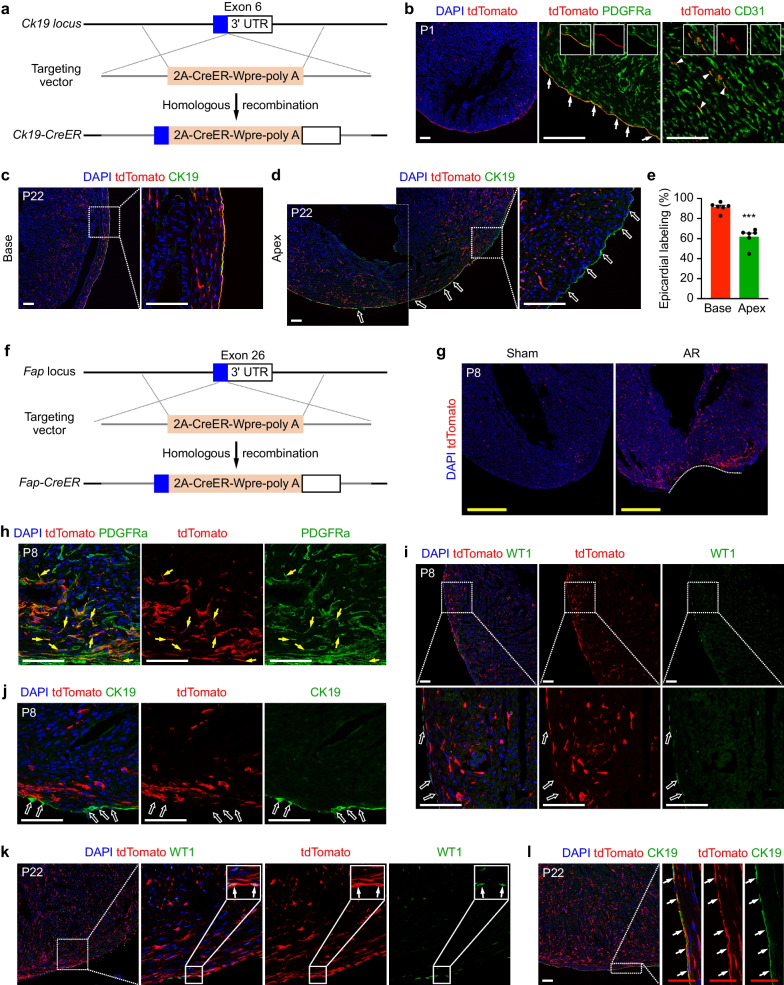
Mesenchymal-to-epithelial transition in hearts after apical resection. **a** Schematic diagram illustrating the approach used to generate *Ck19-CreER* mice. **b** Immunostaining for tdTomato and PDGFRa or CD31 on hearts of *Ck19-CreER;Ai9* mice, which received tamoxifen treatment at E17.5 and harvested for analysis at P1. The arrows indicate the tdTomato-labeled epicardium. The triangles indicate the tdTomato-labeled endothelial cells. Insets indicate colocalization of tdTomato with PDGFRa or CD31. **c**, **d** Immunostaining for tdTomato and CK19 on P22 hearts of *Ck19-CreER;Ai9* mice, which were treated with tamoxifen at E17.5 and subjected to AR at P1. The open arrows in **d** indicate tdTomato^–^ epicardial cells in the apex. **e** The percentage of tdTomato-labeled epicardial cells (tdTomato^+^CK19^+^DAPI^+^/CK19^+^DAPI^+^) in the base and apex at P22 following injury. The data are represented as mean values ± SEM and were analyzed using an unpaired Student’s *t*-test. n = 6 mice per group. ***p < 0.001. Significance was accepted when *p* < 0.05. **f** The strategy for generating *Fap-CreER* mice. **g** tdTomato expression in the sham- or AR-operated hearts of *Fap-CreER;Ai9* mice at P8. The mice were subjected to AR at P1 and treated with tamoxifen at P6. **h**–**j** Immunostaining for tdTomato and PDGFRa, WT1 or CK19 on hearts of *Fap-CreER;Ai9* mice at P8. The yellow arrows in **h** indicate tdTomato-labeled fibroblasts. The open arrows in **i** and** j** indicate tdTomato^–^ epicardial cells. **k**, **l** Immunostaining for tdTomato and WT1 or CK19 on hearts of *Fap-CreER;Ai9* mice at P22. The white arrows in **k** and **l** indicate tdTomato-labeled epicardial cells. White scale bars, 100 µm; Yellow scale bars, 500 µm; Red scale bars, 50 µm. 3′ UTR, 3′ untranslated region; Wpre, woodchuck hepatitis virus posttranscriptional regulatory element; poly A, polyadenylation; AR, apical resection; DAPI, 4′, 6-diamidino-2-phenylindole. E, embryonic day; P, postnatal day. Each picture in **b** and **g**–**i** is representative of 5 individual samples. Each picture in c and d is representative of 6 individual samples

During a study on liver development, robust keratin 19 (CK19) signals, a biliary epithelial marker, were occasionally observed in the outmost layer of the heart at embryonic day (E)17.5 and E18.5 (Additional file 2: Fig. S1a, b). Further co-staining for CK19 and epicardial marker Wilms’ tumor gene 1 (WT1), which is also expressed in coronary endothelial cells (Additional file 2: Fig. S1e, f), confirmed epicardial expression of CK19 at E17.5 and E18.5 (Additional file 2: Fig. S1c, d). To trace these CK19^+^ cells, a *Ck19-CreER* mouse line was generated by knocking the CreER recombinase cDNA into the 6th exon of *Ck19* with a 2A peptide sequence (Fig. 1a). To examine epicardial labeling by *Ck19-CreER*, we crossed *Ck19-CreER* mice with the reporter line *Ai9* (*Rosa26-loxp-stop-loxp-tdTomato*) [3] and treated mice with a dose of tamoxifen at E17.5. The resulting pups showed enriched tdTomato signals in the epicardial layer, with some intramyocardial cells also being tdTomato^+^ (Fig. 1b). Co-staining for tdTomato and fibroblastic/epicardial marker platelet-derived growth factor receptor alpha (PDGFRa) or endothelial marker platelet/endothelial cell adhesion molecule 1 (CD31) revealed that most epicardial cells and a subset of endothelial cells, but not intramyocardial fibroblasts, were tdTomato^+^ (Fig. 1b).

To investigate the fate of epicardial cells during neonatal heart regeneration after injury [4], we performed cardiac apical resection (AR) on *Ck19-CreER;Ai9* pups at P1, which were treated with tamoxifen at E17.5, and examined epicardial labeling by co-staining for tdTomato and CK19 at P22. Interestingly, 91.4 ± 1.90% of the epicardial cells at the base of the heart were tdTomato^+^, while only 61.84 ± 3.60% of the epicardial cells in the heart apex were tdTomato^+^ (Fig. 1c–e). This significant dilution of epicardial labeling in the regenerated apex led us to speculate that there may be a nonepicardial cell lineage contributing to the newly formed apical epicardium during neonatal heart regeneration after AR.

Fibroblast activation protein (FAP) is expressed in cardiac fibroblasts/myofibroblasts after injury but minimally expressed in normal heart tissues [5]. To target fibroblasts in an injured heart, we generated a *Fap-CreER* mouse line through insertion of the CreER cassette into the 26th exon of the *Fap* gene with a 2A sequence (Fig. 1f). Few tdTomato^+^ cells were detected in normal adult hearts of *Fap-CreER;Ai9* mice, which were administered with tamoxifen at postnatal 8 weeks (P8W) and killed after 2 days (Additional file 2: Fig. S2a, b). However, at 7 days after myocardial infarction (MI), a robust tdTomato signal was detected in the infarcted myocardium of adult *Fap-CreER;Ai9* mice, which were administered with tamoxifen at 5 days post-MI (Additional file 2: Fig. S2c). Immunostaining results demonstrated that *Fap-CreER;Ai9* significantly targets PDGFRa^+^ fibroblasts (49.9 ± 1.33%), but not WT1^+^ or CK19^+^ epicardial cells, in the injured cardiac regions post-MI (Additional file 2: Fig. S2d–f).

To investigate whether *Fap-CreER;Ai9* labels fibroblasts in neonatal hearts after AR, we performed sham or AR operations on *Fap-CreER;Ai9* pups at P1, administered a dose of tamoxifen at P6, and analyzed the hearts at P8. We observed more tdTomato^+^ cells in the injured hearts than in the sham-operated hearts (Fig. 1g). Immunostaining showed that *Fap-CreER;Ai9* also labels PDGFRa^+^ fibroblasts but not WT1^+^ or CK19^+^ epicardial cells in the injured hearts (Fig. 1h–j). To investigate whether *Fap-CreER;Ai9*-targeted fibroblasts contribute to de novo epicardial cell formation during heart regeneration, we followed the cell fates of tdTomato^+^ cells until P22. Co-staining for tdTomato and WT1 or CK19 revealed that tdTomato was expressed in epicardial cells at P22 (Fig. 1k, l), suggesting that *Fap-CreER;Ai9*-targeted fibroblasts give rise to epicardial cells during neonatal heart regeneration after AR (Additional file 2: Fig. S3).

Transplantation of epicardium- or epicardial-derived cells is a promising therapy for cardiac repair. Previous studies have shown that transplantation of human epicardial-derived cells into ischemic mouse hearts preserved cardiac function and attenuated ventricular remodeling [6]. Additionally, co-transplantation of human embryonic stem cell-derived epicardial cells and cardiomyocytes improved grafted cardiomyocyte proliferation, increased graft and host vascularization, and promoted cardiac function [7]. However, the safety of pluripotent stem cell-derived cells remains uncertain. Our study revealed that fibroblasts in the injured sites underwent MET to form epicardium during neonatal heart regeneration, suggesting the possibility of directly converting fibroblasts into epicardial cells. This may provide an alternative cell source for transplantation in future epicardial-based regenerative therapies. To our knowledge, this is the first report of MET in vivo for heart regeneration. However, the mechanism underlying MET in heart regeneration is unknown, and further investigations are necessary. This also has important implications for generating fibroblasts-derived epicardial cells in vitro.

## Supplementary Information

**Additional file 1. **Materials and Methods.

**Additional file 2. **Supplementary figures 1–3.

## Data Availability

All data and material generated or analyzed during this study are included in this published article and its additional file.

## Abbreviations

EMT: Epithelial-to-mesenchymal transition
MET: Mesenchymal-to-epithelial transition
AR: Apical resection
FAP: Fibroblast activation protein
MI: Myocardial infarction
CK19: Keratin 19
WT1: Wilms’ tumor gene 1
FAP: Fibroblast activation protein
PDGFRa: Platelet-derived growth factor receptor alpha
CD31: Platelet/endothelial cell adhesion molecule 1, also known as PECAM
