# Supplementary material for: Identification of mesenchymal-to-epithelial transition during heart regeneration through genetic lineage tracing
Source: Stem Cell Res Ther. 2023 Jun 14;14:161. doi: 10.1186/s13287-023-03391-8 (PMC10268380; doi:10.1186/s13287-023-03391-8)
Supplement: Supplementary file 1 — Additional file 1. Materials and Methods. [file 13287_2023_3391_MOESM1_ESM.pdf]

Supplementary Information for:

Identification of mesenchymal-to-epithelial transition during heart regeneration through genetic lineage tracing

Zibei Gao[1, 3], Zhengkai Lu[1, 3], Jinyan Meng[2], Chao-Po Lin[1], Hui Zhang[1, *] and Juan Tang[2, *]

[1]*School of Life Science and Technology, ShanghaiTech University, Shanghai 201210, China.*

[2]*Institute for Regenerative Medicine, Shanghai East Hospital, Frontier Science Center for Stem Cell Research, School of Life Science and Technology, Tongji University, Shanghai 200092, China.*

[3]*These authors contributed equally: Zibei Gao, Zhengkai Lu.*

[*]*Correspondence: Hui Zhang (zhanghui1@shanghaitech.edu.cn); Juan Tang (tangjuan@tongji.edu.cn)*

Materials and Methods:

Experimental mice

All mice used in this study were maintained on a C57BL6/ICR background and were treated in compliance with guidelines of the Institutional Animal Care and Use Committee of ShanghaiTech University. Tamoxifen treatment was administered by oral gavage at the indicated time points, using a dosage of 0.10 mg/g of body weight. The *Ai9* mouse line has been reported previously[1]. The *Ck19-CreER* and *Fap-CreER* mouse lines were generated by the Shanghai Model Organisms Center using CRISPR-Cas9 methods. In the *Ck19-CreER* mouse line, CreER recombinase cDNA was inserted into the 6th coding exon of Ck19, followed by a woodchuck hepatitis virus posttranscriptional regulatory element (Wpre) and a polyadenylation (poly A) sequence. A 2A peptide sequence was used to link the Ck19 coding region and CreER cDNA allowing expression of both Ck19 and CreER. The *Fap-CreER* mouse line was generated using a similar strategy, where the 2A-CreER-Wpre-poly A cassette was inserted into the 26th coding exon of *Fap*, and a 2A peptide sequence was included to enable expression of both Fap and CreER. The mice were housed in a room with a 12-hour light:12-hour dark cycle and provided with free access to sterile food and water *ad libitum*. The room temperature was maintained at 22 °C. The procedures for mice model generation and experiments were conducted in accordance with ARRIVE guidelines.

Details of euthanasia

For neonatal mice born within 10 days (P1 or P8), we use the asphyxiation method using CO_2 followed by decapitation. We followed the guidelines and used a non-precharged chamber for CO_2 asphyxiation. To perform the procedure, the cage cover and wire mesh rack were removed, and a euthanasia lid was placed on the cage. CO_2 was delivered from a pressurized tank to cage. The flow rate was set to displace 30-70% of the chamber or cage volume per minute. After confirming that the mouse had stopped breathing, CO_2 continued to be supplied for more than one minute. Sharp scissors were then used to decapitate the mouse for subsequent experiments. Asphyxiation using CO_2 followed by cervical dislocation was performed on mice older than 10 days old (P22) as well as adult mice. For fetuses (E17.5 or E18.5), we performed cervical dislocation under anesthesia on the pregnant mouse. It was confirmed that there were no signs of life in the mouse and the mouse's uterus was opened and the embryos were removed. The fetuses were immediately decapitated for subsequent experiments.

Apical resection

Apical resection was performed as previously described[2]. At P1, mice were anaesthetized by hypothermia on ice for 3-5 minutes and placed on a frozen operation table once their breathing became steady. The surgical site was disinfected using 70% ethanol and mouse limbs were fixed with forceps. An incision of about 0.5 cm was made, and the sternum vertical of the chest muscles was separated under a stereo microscope. Two or three intercostal incisions were made on the left sternum chest to expose the pericardium and the apex. Curved forceps were extended into the intrathoracic to pull the heart out, and the apex was truncated with microsurgical scissors. Bubbles and blood in the chest were then squeezed out. The chest was sewn up with 8-0 absorbable silk sutures. The skin was sewn up with 11-0 sutures. Post-operation, the mice were placed under a 37°C lamp to keep warm until they woke up. For sham-operated control, the same experimental procedures as above were performed, except for truncating the heart apex.

Myocardial infarction

To induce myocardial infarction, we followed the previously described method of ligating the left anterior descending branch of the coronary artery[3]. The animals were anesthetized via tracheal intubation and administered 2% isoflurane gas while maintaining their core temperature on a 37°C pad. The isoflurane concentration was then adjusted to 1.5% upon reaching a surgical plane of anesthesia. A vertical incision of 1.0-1.5 cm was made at the sternum, and muscle and fascia were bluntly dissected to prevent vessel injury. After making an ~1 cm incision between the third and fourth intercostal ribs, the chest was exposed, and the left anterior descending branch of the coronary artery was permanently ligated with 8-0 sutures. The incisions were sewn up using 6-0 sutures. Following surgery, the cannula was removed, then the mice were kept warm on a 37°C heating plate. The investigators performing surgeries were blinded to the animal group assignments.

Immunostaining

Immunostaining was carried out as previously described[4]. Briefly, frozen sections were dried at room temperature and washed with PBS. The blocking solution was prepared using 5% donkey serum and sections were blocked for 30 minutes at room temperature. Primary antibodies were diluted with a PBS antibody diluent containing 1% Triton X-100 and 2.5% donkey serum, and sections were incubated with primary antibodies overnight at 4°C. The following primary antibodies were used: anti-CK19 (1:500, TROMA-III-S, DSHB), anti-WT1 (1:100, ab89901, Abcam), anti-CDH5 (1:100, AF1002, R&D), anti-PDGFRa (1:200, AF1062s, R&D), anti-CD31(1:250, 553370, BD), anti-tdTomato (1:1000, 600-401-379, Rockland), and anti-MSLN (1:200, ab236546, Abcam). After primary antibody incubation, the sections were washed with PBS and incubated for 1 hour with Alexa Fluor secondary antibodies (Invitrogen) at

room temperature. After washing with PBS, sections were incubated with DAPI for 10 minutes and mounted. Pictures were collected using a laser confocal microscope (Nikon A1R or Zeiss LSM 980).

Statistical analysis

All data were representative of at least three independent experiments and presented as mean values ± SEM. Data for 2 groups were analyzed using an unpaired Student's t-test. Data for 3 groups were analyzed using a 1-way ANOVA followed by Tukey's post hoc test. Significance was accepted when $p < 0.05$. Investigators performing statistics analysis were blinded to all animal group assignments.

References

1. Madisen L, Zwingman TA, Sunkin SM, Oh SW, Zariwala HA, Gu H, Ng LL, Palmiter RD, Hawrylycz MJ, Jones AR *et al*: A robust and high-throughput Cre reporting and characterization system for the whole mouse brain. *Nat Neurosci* 2010, 13(1):133-140.
2. Mahmoud AI, Porrello ER, Kimura W, Olson EN, Sadek HA: Surgical models for cardiac regeneration in neonatal mice. *Nat Protoc* 2014, 9(2):305-311.
3. Jiang Z, Lu Z, Kou S, Feng T, Wei Y, Gao Z, Deng D, Meng J, Lin CP, Zhou B *et al*: Overexpression of Kdr in adult endocardium induces endocardial neovascularization and improves heart function after myocardial infarction. *Cell Res* 2021, 31(4):485-487.
4. Feng T, Gao Z, Kou S, Huang X, Jiang Z, Lu Z, Meng J, Lin CP, Zhang H: No Evidence for Erythro-Myeloid Progenitor-Derived Vascular Endothelial Cells in Multiple Organs. *Circ Res* 2020, 127(10):1221-1232.
