# Supplementary material for: Identification of mesenchymal-to-epithelial transition during heart regeneration through genetic lineage tracing
Source: Stem Cell Res Ther. 2023 Jun 14;14:161. doi: 10.1186/s13287-023-03391-8 (PMC10268380; doi:10.1186/s13287-023-03391-8)
Supplement: Supplementary file 2 — Additional file 2. Supplementary figures 1–3. [file 13287_2023_3391_MOESM2_ESM.pdf]

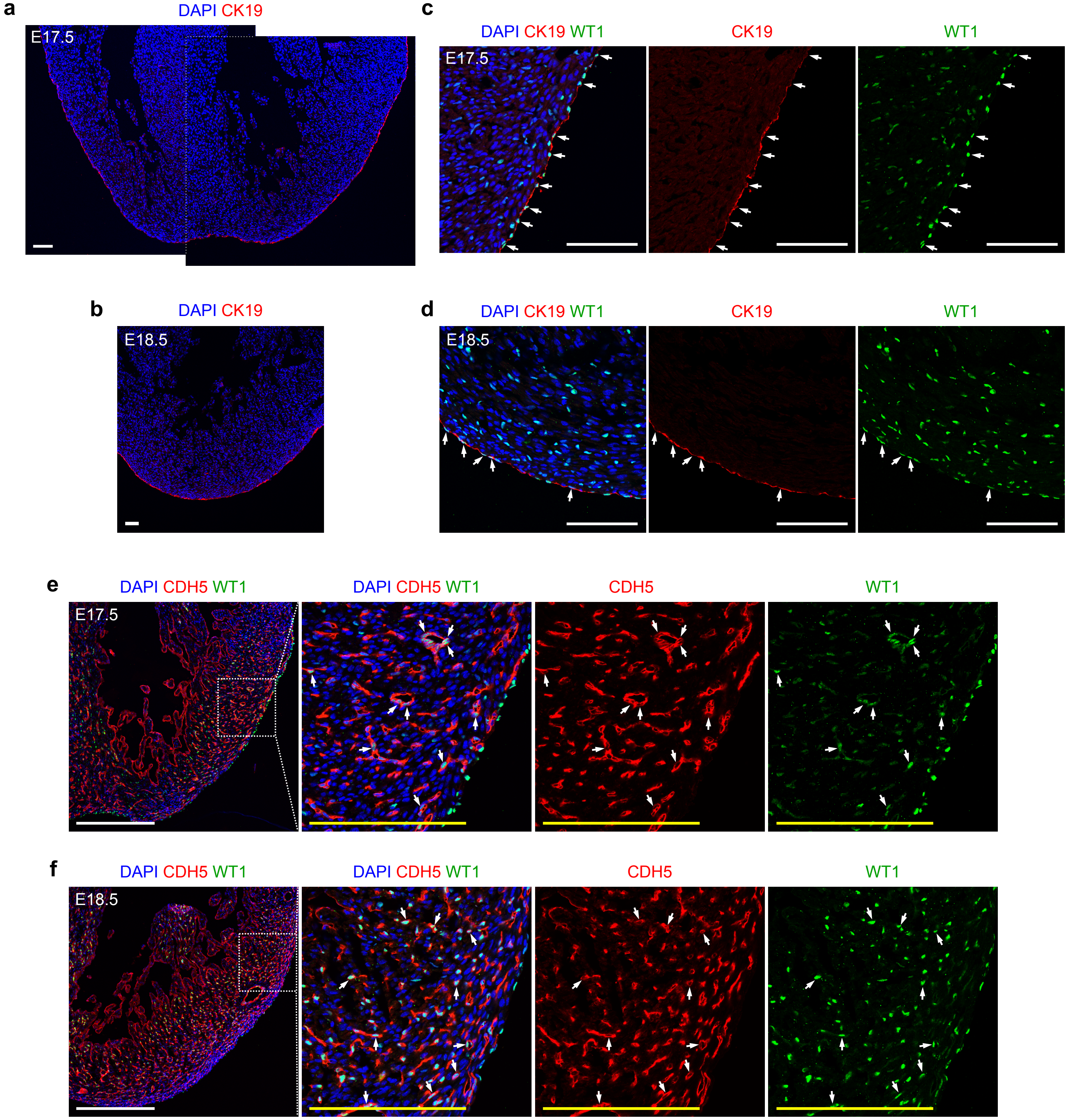

Supplementary information, Figure S1 Expression of CK19 in epicardial cells at late embryonic stages. **(a, b)** Immunostaining for CK19 on hearts at embryonic day (E) 17.5 and E18.5. **(c, d)** Immunostaining for CK19 and WT1 on hearts at E17.5 and E18.5. The arrows indicate CK19$^+$WT1$^+$ epicardial cells. **(e, f)** Immunostaining for CDH5 and WT1 on hearts at E17.5 and E18.5. The arrows indicate CDH5$^+$WT1$^+$ endothelial cells. The boxed regions in **e** and **f** are magnified and split channels on the right. White scale bars, 100 μm; Yellow scale bars, 50 μm. Each immunostaining picture is representative of 5 individual mouse samples.

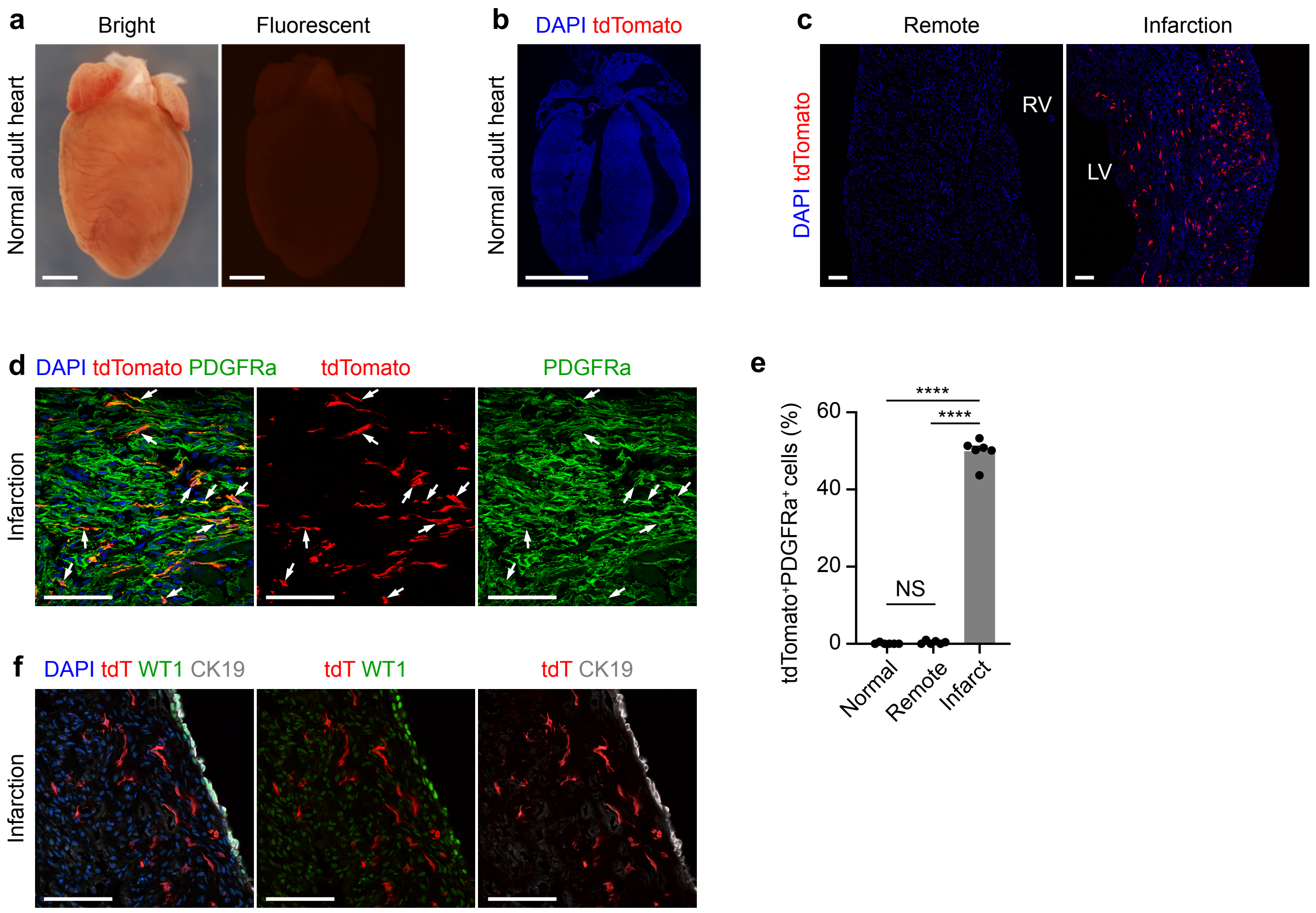

Supplementary information, Figure S2 *Fap-CreER;Ai9* labels fibroblasts, but not epicardial cells, in adult hearts post-MI. **(a)** Whole-mount view of the hearts obtained from adult *Fap-CreER;Ai9* mice, which were administered with a dose of tamoxifen at P8W and sacrificed after 48 hours. Scale bars, 2 mm. **(b)** Few tdTomato signals are detected in the hearts of the P8W *Fap-CreER;Ai9* mice with tamoxifen treatment. Scale bar, 2 mm. **(c)** tdTomato is robustly detected in the infarcted regions of *Fap-CreER;Ai9* mice, which were treated with tamoxifen at 5 days post-MI and harvested at 7 days post-MI. RV, right ventricle; LV, left ventricle. Scale bars, 100 μm. **(d)** Immunostaining for tdTomato (tdT) and PDGFRa on hearts of *Fap-CreER;Ai9* mice at 7 days post-MI. The arrows indicate tdTomato-labelled PDGFRa$^+$ cells. Scale bars, 100 μm. **(e)** The percentage of tdTomato-labelled PDGFRa$^+$ cells in the normal and injured hearts. n = 6 samples per group. NS, non-significant; **** $p < 0.0001$. **(f)** Immunostaining for tdTomato, WT1 and CK19 on hearts of *Fap-CreER;Ai9* mice at 7 days post-MI. tdT, tdTomato. Scale bars, 100 μm. Each immunostaining picture is representative of 6 individual mouse samples.

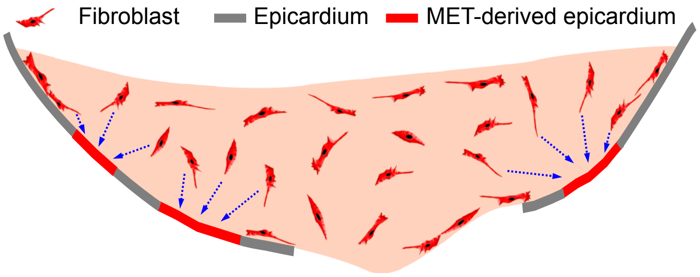

Supplementary information, Figure S3 Schematic diagram showing MET in the injured region during neonatal heart regeneration after AR. MET, mesenchymal-to-epithelial transition.
